# Perspectives and preferences on palliative chemotherapy-immunotherapy in gallbladder cancer patients

**DOI:** 10.3332/ecancer.2026.2111

**Published:** 2026-04-09

**Authors:** Koreddi A Dora, Vaka Rajashekhar, Mahadevapura R Kaushik, Gaurav Vora, Arti Sarin, Amol Patel

**Affiliations:** 1 Department of Medicine, Indian Naval Hospital Ship, Asvini, Colaba, Mumbai 400005, India; 2 Department of Radiation Oncology, Indian Naval Hospital Ship, Asvini, Mumbai 400005, India; 3 Director General Armed Forces Medical Services, Delhi 110023, India; 4 Malignant Disease Treatment Centre, Army Hospital Research and Referral, Delhi 110010, India

**Keywords:** palliative chemotherapy, gallbladder cancer, cholangiocarcinoma, NCCN distress thermometer, quality of life

## Abstract

**Background:**

Patients’ perspective and preferences about the chemotherapy are not known. This study investigates the perspectives and treatment preferences of Indian gallbladder (GBC) patients.

**Methods:**

A prospective 18-month study at a tertiary cancer center in Mumbai enrolled patients with histologically confirmed GBC receiving palliative chemotherapy, who were counselled in a structured format regarding disease, treatment options, outcomes and drug costs. Data were collected through structured interviews using quality-of-life and decision-making questionnaires. Descriptive statistics were used to analyse patient responses. A pilot sample of 30 patients was used to assess whether ≥90% prioritise survival as the primary expectation from chemo-immunotherapy. Using an exact one-sided binomial design, ≤23 responses favouring survival would indicate the proportion is <90% and warrant further study. We also evaluated the psychological distress among these patients.

**Results:**

Among 30 participants (56.67% female), 83.33% were of advanced stage. Baseline demographics is depicted. Treatment priorities varied: 40% preferred life prolongation, 23.33% favoured symptom relief and 36.67% valued both equally. Financial burden strongly influenced decisions about immunotherapy, only 6.67% were willing to pay ₹35 lakhs out-of-pocket for a 1.5-month gain, while 100% would take treatment if it were government-funded. Emotional distress was significant, with a mean NCCN score of 6.21 ± 1.80. Major concerns included financial strain (42%), fear (45%) and depression (33%).

**Conclusion:**

Twenty-three percent of patients favoured only symptom relief as a treatment when they were educated about the outcomes. A larger study is warranted to further consolidate our findings.

## Introduction

Gallbladder (GBC) cancer is one of the most aggressive malignancies of the biliary tract, known for its poor prognosis and wide geographical variation in incidence. It is particularly prevalent in Northern India, Chile and East Asia, likely due to environmental, dietary and genetic risk factors [1]. In India, it is the most common gastrointestinal cancer among women, especially in northern states [2]. Despite advancements in imaging and pathology, it often presents at an advanced stage, making curative resection possible in <10% of cases [3]. The current standard of care includes gemcitabine-based regimens, particularly the combination of gemcitabine and cisplatin or oxaliplatin, which demonstrated a modest survival benefit in the landmark ABC-02 trial [4, 5]. However, even with optimised chemotherapy, median overall survival in advanced biliary tract cancers rarely exceeds 6–9 months and 5-year survival remains dismissal. Trials investigating intensified regimens with oxaliplatin, nab-paclitaxel and immunotherapy agents like durvalumab have shown minimal incremental benefits [6, 7]. The cost of immunotherapy forbids the access to the majority of patients [8].

Literature from high-income settings has shown that many patients receiving palliative chemotherapy believe it to be curative, despite communication of its intent as life-prolonging or symptom-relieving [9]. In cancers such as lung, breast and colorectal, this misunderstanding has led to unrealistic expectations, treatment dissatisfaction and compromised end-of-life planning. Whether this mismatch exists in GBC cancer, particularly in resource-limited, high-incidence regions like India, remains largely unexplored. Equally important yet under-recognised is the emotional burden experienced by patients undergoing palliative chemotherapy. Distress manifesting as anxiety, depression, fear or uncertainty has been associated with poorer adherence to treatment, higher symptom burden and reduced quality of life [10]. Structured tools such as the NCCN Distress Thermometer can aid in quantifying this burden, but are rarely integrated into routine oncology care.

Understanding patient perspectives is essential not only for promoting shared decision-making but also for ensuring ethically sound, culturally appropriate and patient-centered care in high-burden, resource-constrained settings. This study aims to systematically assess the expectations, treatment priorities, willingness to accept risk and psychosocial burden among patients undergoing palliative chemotherapy for GBC cancer.

## Methods

This was a prospective observational study conducted in the Department of Medical Oncology at a tertiary care hospital in Western India with a primary objective to estimate patient perspectives, preferences regarding palliative chemotherapy and determine patients’ understanding of survival benefit expectations. We also evaluated emotional distress and psychosocial burden using the NCCN thermometer. The study was carried out over a period of 18 months. The study was approved by the Institutional Ethics Committee prior to commencement.

The study included patients who were above 18 years of age and had a histologically confirmed diagnosis of GBC carcinoma. These participants had either been planned for or were undergoing palliative chemotherapy at the time. Inclusion also required that the patients were able to understand and respond to the study questionnaire in Hindi, Marathi or English. Patients were excluded if they lacked a histological diagnosis or exhibited cognitive impairments that rendered them unable to comprehend and respond to the structured questions.

The collection of data were done by the interview method. After assuring confidentiality and obtaining written informed consent, the patients were counselled systematically (Appendix 1) and briefed about the treatment benefits, outcomes and cost. After this, the questionnaire (Box 1) was explained to the participant. The answers were recorded in paper format and later exported to Excel. The replies of the participant to NCCN distress thermometer were also recorded. Participant information sheet and informed consent form in English, Hindi and Marathi languages were used. Data were entered into Microsoft Excel and analysed using SPSS version 25.0. Descriptive statistics were used to summarise demographic variables and questionnaire responses. Findings were mentioned in frequency and percentages.

### Statistics

The primary objective of this study is to estimate the percentage of patients opting for increase in survival as the primary expectation from chemotherapy and immunotherapy. If that percentage is <90%, then additional studies might be warranted. As this is a pilot, a sample size of 30 would be adequate. If ≤23 of 30 patients opt for increase in survival as the primary expectation from chemotherapy, the upper bound of the one-sided 95% confidence interval for the proportion of patients who opt for an increase in survival as the primary expectation from chemotherapy will be <90% and additional studies might be warranted.

Box 1. **Patient questionnaire**

**According to you which of the following is the most important**
  - Decrease in symptoms
  - Prolongation of Life
  - Both of the above
  - Something else ( specify ...........)
**If it is both of the above which is more important?**
  - Decrease in symptoms even if life is not prolonged
  - Prolongation of life even if symptoms and problems do not decrease
  - Both have equal importance
**3.According to you prolonging life means prolonging life by at least prolonging life by**
  - 1-month
  - 2-4 months
  - 4-6 months
  - > 6 months-1 year
  - > 1 year
**As explained to you earlier, you would have realised that chemotherapy will not prolong life of all patients; If so: If how many people benefit will you be willing to take chemotherapy?**
  - One person in 100
  - One person in 50
  - One person in 20
  - One person in 10
  - One person in 5
**As explained to you earlier, you would have realised that chemotherapy will not decrease symptoms and problems of all patients; If so: If how many people benefit will you be willing to take chemotherapy?**
  - One person in 100
  - One person in 50
  - One person in 20
  - One person in 10
  - One person in 5
**What percentage (%) of your lives do you wish to spend in each of the following places shown in the picture below**
  - home
  - hospital
  - work place
**You would have understood that as a result of side effects of chemotherapy there is a risk of dying. Upto what % are you ready to accept the risk of death due to toxicity?**
  - a) 1% risk
  - b) 2%-5% risk
  - c) 6%-10% risk
  - d) 11%-20% risk
**Crtain side effects of chemotherapy affect day to day living activities. Upto what % are you ready to accept such side effects?**
  - a) 1%-10% risk
  - b) 11%-20% risk
  - c) 21%-30% risk
  - d) 31%-40% risk
**Number the following side effects from 1 to 10 in ascending order of their acceptability to you, that is 1st been most unacceptable and 10th been most acceptable.**
  - a) Fever
  - b) Vomiting
  - c) Nausea
  - d) Loose motions
  - e) Skin rashes
  - f) Numbness of hands and feet
  - g) Muscle pain
  - h) fatigue
  - I) Weakness of body
  - J) constipation
**If you require hospital admissions for side effects upto how many times minimum it is acceptable to you?**
  - a) zero times/ No admission
  - b) 1-2
  - c) 3-4
  - d) > 5 times\
**To improve your disease control are you ready to participate in a new drug study?**
  - a) Yes
  - b) No. If No why no.
**Would you like to take treatment if your life is increased by 01 month if new medicine is added to existing standard of care?**
  - a) Yes
  - b) No
**For adding this 1.5 month life, the total expenditure is 35 lakhs. Would you still want this treatment?**
  - a) Yes
  - b) No
**If answer is Yes to previous question, if you have to pay this money from your pocket, would you still consider this treatment**.
  - a) Yes
  - b) No
**If answer is Yes to previous question, if treatment cost is sponsored by Government, would you still consider this treatment.**
  - a) Yes
  - b) No

## Results

The baseline demographics are depicted in Table 1. Symptomatic relief to chemotherapy was reported by 17 patients (56.67%), indicating subjective benefit from palliative chemotherapy. However, adverse effects during chemotherapy were reported by 21 individuals, showing a high incidence of treatment-related morbidity.

For diverse treatment preferences and survival expectations among participants when asked about primary treatment goals, 40% preferred life prolongation, 23% prioritised symptom relief and 37% valued both equally. Among the subgroup (*n* = 11) who considered both important,

six patients still favoured life extension over symptom control. With regard to meaningful life expectancy gain, half of the participants felt 6–12 months was worthwhile, while 37% expected more than a year. The primary objective was met as 23% patients would like to adopt for symptom control over palliative chemotherapy-immunotherapy.

While all 30 patients were willing to accept a new drug if it extended life by 1 month, only 7% agreed to pay ₹35 lakhs for a 1.5-month gain. The same fraction (7%) accepted the treatment when required to pay out-of-pocket. However, if the cost was covered by the government, all participants unanimously agreed to receive the treatment.

In the risk thresholds and side-effect tolerance levels, 43% of participants accepted a 1% risk of death due to chemotherapy and another 27% accepted up to 5%. Regarding tolerable side-effect rates, 43% accepted a 31%–40% risk. Vomiting was considered the most unacceptable adverse effect by 37% of patients, followed by fever (17%) and muscle pain, nausea or weakness (13% each). Conversely, 43% identified skin rashes as the least concerning, followed by loose stools and fatigue (20% each). When evaluating acceptable hospitalisation, 40% accepted 1–2 admissions, 30% were fine with 3–4 visits and 23% preferred no hospital stay at all.

NCCN Distress Scale and associated checklist’s findings are shown in Table 2. The mean distress score was 6.21 ± 1.80, suggesting a moderate to high level of emotional stress. Practical problems were led by financial concerns (42.42%), followed by difficulty in treatment decisions (18.18%). Family-related issues were present in 40% of patients, mainly involving spouses and children. Emotional distress was high, with 46% reporting fear and 33% reporting depression. Spiritual or religious concerns were present in nearly a quarter (24%). On the physical symptom checklist, pain (27.27%), fatigue (24.24%), nausea (18.18%) and sleep disturbances (18.18%) were the most common, while 12% noted issues related to body image or appearance.

Half of the participants preferred to spend their remaining life at home, 33% at the workplace and only 17% in a hospital setting. Participation in a new drug trial was low, with only 14% willing to enroll.

## Discussion

In a seminal work by Weeks *et al* [9], 69% and 81% lung and colon cancer patients, respectively, did not understand the true nature of chemotherapy benefits, meaning patients in palliative care setting do feel that chemotherapy would cure them. We echoed the finding of study by Patil *et al* [11] in head and neck cancer wherein the symptom control was preferred by almost 60% of patients. We did not find similar study done previously in biliary tract cancers.

Koedoot *et al* [12] did somewhat in a different way that, they analyse the preferences of chemotherapy in a palliative setting before meeting the medical oncologist. Additional 10% of patients adopted chemotherapy to baseline pre-consultation rate of 68%. Again, 22% of patients would not opt for chemotherapy in Amsterdam. Exactly, we had a similar rate of not opting of palliative chemotherapy in Indian GBC patients.

The role of cost is of paramount importance. While 100% of patients were willing to undergo treatment if it added 1.5 months of life, only 7% accepted the same benefit at an out-of-pocket cost of ₹35 lakhs. When fully subsidised by government schemes, acceptance rose again to 100%. This finding demands further research for cost-effectiveness studies and willing to pay threshold in the Indian setting to optimise resources with the focus on improving access rather than denying treatment [13].

Emotional distress was a prominent concern. The mean NCCN distress score was 6.21, indicating moderate-to-severe distress. Financial strain was the most cited practical issue (42.42%), followed by difficulty in treatment decision-making (18.18%). Emotional symptoms such as fear (45.45%) and depression (33.33%) were widespread. Temel *et al* [14] showed that early palliative care mitigates such distress and improves emotional resilience and quality of life. Spiritual concerns and physical symptoms (pain, fatigue, nausea and sleep problems) also added to the psychological burden, further justifying the need for integrative psychosocial support.

Our study is of particular importance in various aspects; we have brought certain aspects forward in terms of patients’ perceptions as it put emphasis on policies of patient autonomy and participation in decision making, which might make the difference, at least for those 25% of patients who do not want toxic chemotherapies and costly immunotherapies. We also brought a very other important aspect, almost 85% patients are not willing to spend time in hospital, which are consistent with the reports by Leng *et al* [15]. Time toxicity is as important as other toxicities of adverse events and financial toxicities. The time spent by the patients with treatment that offers modest survival benefit is not just about the 2 hour infusion, but before and after that, time spent for blood draws, scans, waiting time at OPD, travel and hospitalisation required for higher grade toxicities. As suggested by Gupta *et al* [16], the time toxicity measures must be included in clinical trials and we strongly recommend it.

This study was conducted at a single tertiary care institution, which may limit the generalisability of the findings to broader populations or diverse healthcare settings. The other limitation is limited number. However, as per our hypothesis considered on the basis of previous reports, the 23% of preference to only symptom relief is replicated. For deriving policies, further large studies on other cancer types needs to be started on priority.

## Conclusion

One quarter of patients want only symptom control when the benefits of chemotherapy are limited. Though many patients prioritised life prolongation, their choices were constrained by significant emotional distress. Larger studies are warranted to integrate psychosocial support and adopting a patient-centered, preference-based approach beyond clinical metrics, which can enhance treatment satisfaction and quality of life.

## Conflicts of interest

None.

## Funding

No funding received for the study.

## Figures and Tables

**Table 1.** Baseline demographics.

Characteristic	Category	Frequency	Percentage (%)
Gender	Female	18	56.67
	Male	12	43.33
Age	52 years (range: 23–74)		
Marital status	Married	25	83.33
	Unmarried	5	16.67
Occupation	Not-employed	14	46.67
	Retired	8	26.66
	Working	8	26.67
Residence	Rural	9	30.00
	Urban	21	70.00
Symptomatic relief	Present	17	56.67
	Absent	13	43.33
Adverse effects of chemotherapy	Present	21	70.00
	Absent	9	30.00

**Table 2.** NCCN distress scale and NCCN Scale

NCCN distress scale	Mean	Standard deviation
	6.21	1.80
**NCCN scale**	**Frequency**	**Percentage**
Practical problems		
Financial	14	42.42
Treatment decisions	06	18.18
Nil	13	39.39
Family problems		
Dealing with partner	8	24.24
Dealing with children	5	15.15
Nil	20	60.61
Emotional problems		
Depression	11	33.33
Fears	15	45.45
Nil	7	21.21
Spiritual/religious concerns		
Present	8	24.24
Absent	25	75.76
Physical problems		
Pain	9	27.27
Fatigue	8	24.24
Nausea	6	18.18
Sleep	6	18.18
Appearance	4	12.12

## Appendix 1. Sociodemographic survey.

- **Stage of disease**
- **Incurable nature**
- **Prognosis:** Median OS without chemotherapy 2-3 months and 1 year survival below 10%, Median OS with chemotherapy 8 months, 1 year survival 10%-20%, Median OS gain with Durvalumab-based immunotherapy plus chemotherapy is 1.3 months, 2 year survival rate 24% for cholangiocarcinoma, exact figure for GBC cancer is not known.
- **Relief in symptoms**: Pain relief seen in nearly 60%-70% of patients. The symptoms would decrease subjectively in 50%-60% of patients.
- **Side effects of chemotherapy**
  1. **Durvalumab-based (TOPAZ study): Chemotherapy plus immunotherapy**
    - Any grade side effects: Cumulative percentage 80%-100% (Common adverse events: anorexia, myalgia, allergic reactions, rash, diarrhea, fatigue, neuropathy)
    - Grade 3-4 side effects: Cumulative percentage 35%-40% (Serious common adverse events: febrile neutropenia, grade 3-4 diarrhea, grade 3-4 rash, grade 3 fatigue, electrolyte imbalances, grade 3-4 neuropathy)
    - Death due to side effects: 2 %
  2. **Only chemotherapy (Gem-Cis) existing standard**
    - Any grade side effects: Cumulative percentage 60%-80% (Common adverse events: anemia, fatigue and mucositis)
    - Grade 3-4 side effects: Cumulative percentage 15%-20% (Serious common adverse events: febrile neutropenia, grade 3-4 mucositis, grade 3 fatigue, grade 3 fatigue, pneumonia)
    - Death due to side effects: <1%
- **Routes of administration:**
- **Duration of treatment: till intolerable side effects or progression of disease**
- **Cost as discussed above**
  1. Durvalumab-based: 20 lakhs Rs/6 months of treatment
  2. Gem-Cis: 50,000 Rs/6 months of treatment
- **Social support requirement**
  1. Need to stay in Mumbai/nearby place approachable to the hospital within 2-3 hours.
  2. Family support
- **Warning symptoms**
  1. If any of the following occurs the patients should report to INHS ASVINI if taking chemotherapy with us or with the treating physician if opts for treatment outside
    - Fever ( temperature 100°F or more)
    - More than three vomitings or loose motions
    - Oral ulcers making it difficult for taking solid foods
    - Extreme fatigue or dizziness
    - Any issues which the patient feels is serious
  2. If, for any reason, they are unable to come to the hospital then they should report to local Physician for care
- **Advice regarding diet**
  1. No outside eatables.
  2. Anorexia would happen hence to take small regular diets.
